# Analysis of the Recurrence of Adverse Drug Reactions in Pediatric Patients with Epilepsy

**DOI:** 10.3390/ph18081116

**Published:** 2025-07-26

**Authors:** Ernestina Hernández García, Brenda Lambert Lamazares, Gisela Gómez-Lira, Julieta Griselda Mendoza-Torreblanca, Pamela Duke Lomeli, Yessica López Flores, Laura Elena Rangel Escobar, Eréndira Mejía Aranguré, Silvia Ruiz-Velasco Acosta, Lizbeth Naranjo Albarrán

**Affiliations:** 1 Laboratorio de Farmacología, Subdirección de Medicina Experimental, Instituto Nacional de Pediatría, Ciudad de México 04530, Mexico; ernestina_hg@aol.com (E.H.G.); pamu1242@gmail.com (P.D.L.); yess_lo@outlook.com (Y.L.F.); lauraes89@hotmail.com (L.E.R.E.); 2 Programa de Maestría y Doctorado en Ciencias Matemáticas y de la Especialización en Estadística Aplicada, Instituto de Investigaciones en Matemáticas Aplicadas y en Sistemas, Universidad Nacional Autónoma de México, Ciudad de México 04510, Mexico; brendalambertlamazares@gmail.com; 3 Departamento de Farmacobiología, Centro de Investigación y Estudios Avanzados del Instituto Politécnico Nacional, Ciudad de México 14330, Mexico; glira@cinvestav.mx; 4 Laboratorio de Neurociencias, Subdirección de Medicina Experimental, Instituto Nacional de Pediatría, Ciudad de México 04530, Mexico; 5 Departamento de Innovación Médica, Hospital Regional de Alta Especialidad de Ixtapaluca, Servicios de Salud del Instituto Mexicano del Seguro Social para el Bienestar, Estado de México 56530, Mexico; innovacion.hraei@gmail.com; 6 Departamento de Probabilidad y Estadística, Instituto de Investigaciones en Matemáticas Aplicadas y en Sistemas, Universidad Nacional Autónoma de México, Ciudad de México 04510, Mexico; silvia@sigma.iimas.unam.mx; 7 Departamento de Matemáticas, Facultad de Ciencias, Universidad Nacional Autónoma de México, Ciudad de México 04510, Mexico

**Keywords:** recurrence regression models, risk factors, adverse drug reactions, epilepsy, anti-seizure medication, seriousness

## Abstract

Epilepsy is a chronic neurological disease with a relatively high incidence in the pediatric population. Anti-seizure medication (ASM) may cause adverse drug reactions (ADRs), which may occur repeatedly. **Objective:** This study aimed to analyze the recurrence of ADRs caused by ASMs over a period of 122 months in hospitalized Mexican pediatric epilepsy patients. The patients were under monotherapy or polytherapy treatment, with valproic acid (VPA), phenytoin (PHT), and levetiracetam (LEV), among others. A total of 313 patients met the inclusion criteria: 211 experienced ADRs, whereas 102 did not. Patient sex, age, seizure type, nutritional status and related drugs were considered explanatory variables. **Methods:** Four statistical models were used to analyze recurrent events that were defined as “one or more ADRs occurred on a single day”, considering both the classification of ADR seriousness and the ASM causing the ADR. **Results:** A total of 499 recurrence events were identified. The recurrence risk was significantly greater among younger patients for both nonsevere and severe ADRs and among those with focal seizures for nonsevere ADRs. Interestingly, malnutrition was negatively associated with the risk of nonsevere ADRs, and obesity was positively associated with the risk of severe ADRs. Finally, LEV was associated with a significantly greater risk of causing nonsevere ADRs than VPA. However, LEV significantly reduced the risk of severe ADRs compared with VPA, and PHT increased the risk in comparison with VPA. In conclusion, this study offers a robust clinical tool to predict risk factors for the presence and recurrence of ASM-ADRs in pediatric patients with epilepsy.

## 1. Introduction

Epilepsy is a brain condition that causes recurrent seizures in children and is one of the most common and disabling neurological disorders [1]. Approximately 1 in 150 children are diagnosed with epilepsy during the first 10 years of life, with the highest incidence rate observed during infancy [2]. The clinical manifestations of epilepsy are highly variable, depend on the cortical area affected, and may include alterations in consciousness, motor function, and sensory perception [3,4]. The International League Against Epilepsy (ILAE) proposed a classification that presents three levels of organization, starting with the type of seizure (focal, generalized, or of unknown origin), the next step is the diagnosis of the type of epilepsy, and the third level is the epileptic syndrome, which refers to a set of features that tend to occur together [5]. Specialists can compile all the information obtained from the previous multi-levels and thus determine the appropriate treatment for patients.

The goal of epilepsy treatment is to control, stop, or reduce the frequency of seizures. This treatment is most often prescribed with anti-seizure medications (ASM), which are selected on the basis of the seizure type, the child’s age, side effects, cost, and ease of use [1]. Although more than 20 clinically used ASM act through a variety of mechanisms of action, all work to reduce neuronal hyperexcitability by either decreasing excitatory or enhancing inhibitory neurotransmission [6]. Like any other medication, ASM are able to cause adverse drug reactions (ADRs), which are harmful, unintended responses to a medicine, occurring at doses usually used for treatment [7]. Common ASM-ADRs include headaches, fatigue, dizziness, drowsiness, blurred vision, nausea, and weight gain or loss [8,9]. More serious reactions, although rare, can include Stevens–Johnson syndrome, liver failure, and pancreatitis [10]. Moreover, adverse psychiatric effects include depression, anxiety, irritability, impaired concentration and mood changes [9,11]. In children and adolescents, common ASM-ADRs are mainly skin rashes, gastrointestinal disturbances, somnolence and behavioral problems [12,13]. Particularly in Mexican children, drowsiness, irritability, alopecia, and thrombocytopenia are the most common adverse effects [9].

ASM-ADRs can be classified by frequency, symptoms, organ, and system affected, as well as severity (mild, moderate, and severe) and seriousness (severe and nonsevere) [9,14,15]. In our previous study, Hernández et al. (2023) reported that, in hospitalized Mexican pediatric epilepsy patients, therapy and nutritional status were risk factors associated with the seriousness of ASM-ADRs in children [9]. The results revealed a greater probability of presenting severe ADRs among patients treated with more than one ASM, reporting probabilities of 1.84 and 2.11 times higher for polytherapy than for monotherapy in the univariate and multivariate analyses, respectively. For nutritional status, severe and moderate malnutrition had probabilities of 1.67 and 2.06 times greater, respectively, of presenting severe ADRs than did normal weight in the multivariate analysis. In addition, levetiracetam (LEV) provoked more nonsevere ADRs, phenytoin (PHT) provoked more severe ADRs, and valproic acid (VPA) caused similar percentages of severe vs. nonsevere ADRs. Moreover, most severe ADRs were preventable, whereas nonsevere ADRs were nonpreventable [9]. These results have been useful in understanding the safety and efficacy of drugs in patients.

Furthermore, ASM-ADRs may be evaluated as recurrent events, which are events that may occur more than once over the follow-up time for a given subject. Recurrent event models offer a more comprehensive and realistic approach for analyzing data where individuals may experience multiple events over time. Unlike traditional models that focus only on the time to the first event, they make use of the full event history, increasing statistical power and providing better insights and estimates. These models also account for within-subject correlation and time-varying risks, making them particularly useful in evaluating long-term patient susceptibility to repeated adverse events. Therefore, the objective of the present study was to analyze the recurrences of ASM-ADRs with the highest prescription rates in hospitalized Mexican pediatric patients with epilepsy at the National Institute of Pediatrics (INP) from October 2012 to December 2022.

## 2. Results

### 2.1. Number of Recurrences of ASM-ADR

Table 1 shows the frequency and distribution of patients by the number of ASM-ADR recurrence events for each explanatory variable. Among a total of 313 children, 211 with recurrences were observed. This means that 102 children did not present ASM-ADRs, 91 children presented only one ASM-ADR event, 55 children presented two events, and so on, up to cases in which 1 child presented ten events, 1 child presented eleven events, and, finally, 1 child presented twelve ASM-ADRs events. Importantly, in this study, a recurrent event is defined as one in which “one or more ADRs occur in one day”, taking into account the classification of ADR seriousness (nonsevere, severe or ADR absence) and the drug causing the ADR (VPA, LEV, PHT, oxcarbazepine (OXC), topiramate (TPM), or other). To examine the relationship between the number of recurrent ADR events and each of the variables, the recurrences were considered discrete variables, and the explanatory variables were categorical.

### 2.2. Number of Recurrences of ASM-ADR by Seriousness

**Figure 1:**
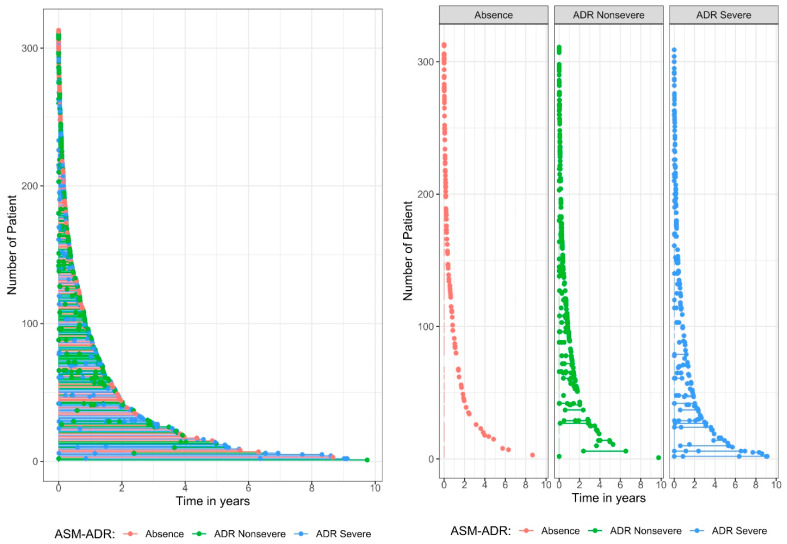
Plot of recurrent events per patient by adverse drug reaction (ADR) seriousness.

Figure 1 displays the plots of recurrence events per patient according to ADR severity. The graphs show the time at which the ADR event occurred, colored by the type of ADR seriousness. Note that each child starts at time = 0, which is the date they begin treatment. When the ADR seriousness events are compared between severe and nonsevere events, those classified as severe recurrences occur over a long period of time.

Table 2 shows the distribution of ASM-ADR by seriousness. The frequencies were obtained by year of occurrence, which means that 0–1 year refers to events that occurred between the start of treatment and before the end of the year (time between 0 and <1 year), 1–2 year refers to events that occurred between years 1 and <2 years, and so on. The results revealed that ADRs occurred most frequently in the first year after the start of treatment, with a frequency of 352 among the total of 499, which means that 70.5% of the ADR events occurred in the first year after the start of treatment. When ADR seriousness events are compared between severe and nonsevere ADRs, severe ADRs occur over a long period of time, whereas nonsevere ADRs occur less frequently over the long term.

### 2.3. Results of the Kaplan–Meier Survival Function Estimate

**Figure 2:**
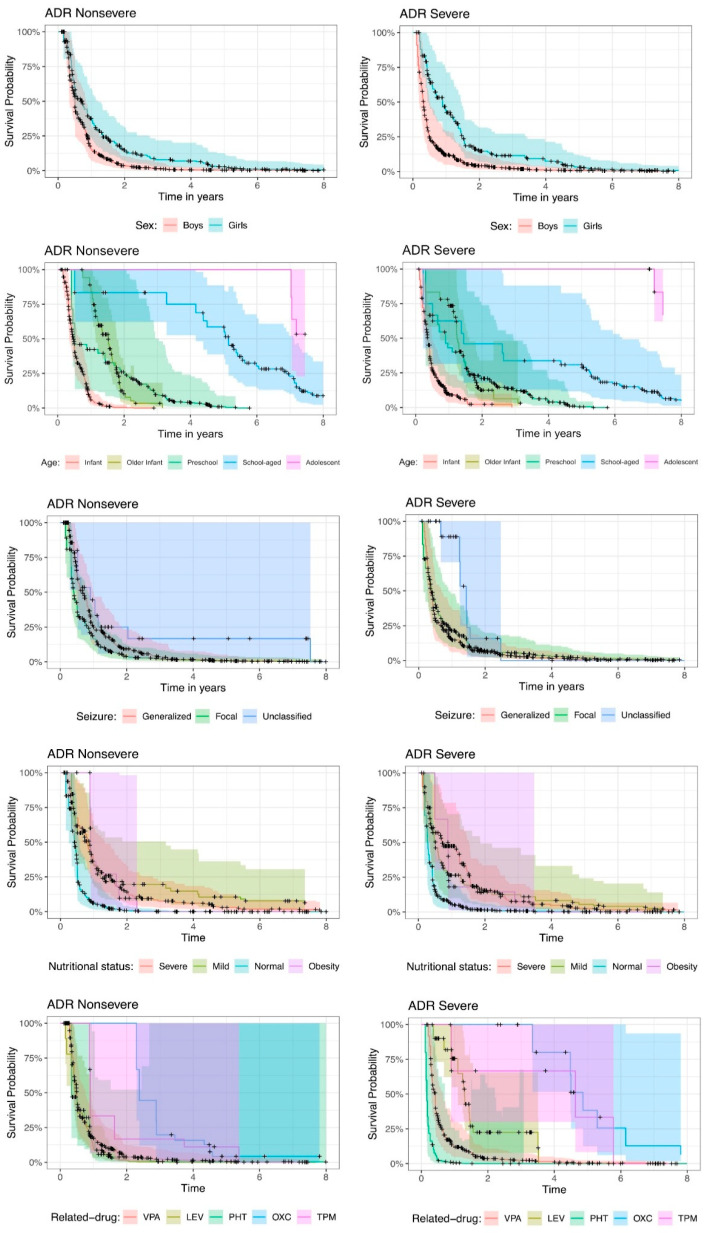
Kaplan–Meier survival function estimates per explanatory variable by Adverse Drug Reactions (ADR) seriousness. VPA: valproic acid; LEV: levetiracetam; PHT: phenytoin; OXC: oxcarbazepine; TPM: topiramate.

Figure 2 shows the Kaplan–Meier survival function estimate, by ADR seriousness, ADR nonsevere or ADR severe, per explanatory variable. The bold lines plot the Kaplan–Meier survival function estimate; the cross dots represent the censors, i.e., the absence of ADRs; the shades on the graphs provide the 95% pointwise confidence intervals for the fitted data. The confidence intervals allow us to detect significant variables, that is, to identify whether there is a statistically significant difference between the categories of each variable.

In the case of the sex of children, there is no significant difference between boys and girls since the confidence intervals intersect at all times. Although the risk of ADR recurrence events is greater in boys than in girls, this difference is not statistically significant.

The age of the children is statistically significant, since the confidence intervals do not always intersect, and there are points in time where the intervals are completely separated for different categories, showing differences between age categories. This occurs primarily in nonsevere ADRs. For severe ADRs, the intervals intersected more often, with fewer differences according to age. In general, infants have the highest risk of experiencing ADR recurrence. For example, for nonsevere ADRs, for infants, the probability of having experienced an ADR in the first year is approximately 94%, whereas for severe ADRs, the probability is 91%. Compared with school-age children, for both nonsevere and severe ADRs, the probabilities of having experienced an ADR in the first year are 17% and 37%, respectively. Note that for the same year equal to 1, the confidence intervals for infants and school-age children are completely separate, indicating a significant difference.

According to the classification of seizures, the significant differences change according to ADR seriousness. Compared with generalized seizures, nonsevere focal seizures present a greater risk of ADR recurrence, although this difference is not statistically significant since the confidence intervals overlap. For severe ADRs, the risk of ADR recurrence between focal and generalized seizures is similar, and unclassified seizures present a lower risk of ADR recurrence.

The nutritional status at baseline is a significant variable, with significant differences between some categories; that is, the confidence intervals do not intersect at various points in time. For nonsevere ADRs, mildness and obesity appeared to be the categories with the lowest probability of ADR recurrence. For severe ADRs, severe and mild ADRs are the categories with the lowest probability of ADR occurrence.

With respect to the drugs that cause ADRs, some drugs show statistically significant differences, but the risk or protection associated with these drugs changes according to the seriousness of the condition. Nonsevere ADRs are associated with a greater risk of ADR recurrence under VPA, LEV and PHT, with similar probabilities. In severe ADRs, PHT is associated with a greater risk of ADR recurrence than VPA, and LEV is associated with a lower risk of ADR recurrence than VPA.

### 2.4. Results of the Recurrent Events Regression Models

Table 3 shows the risk factors associated with ASM-ADR recurrence events in patients with nonsevere seriousness according to sex, age, seizure type, nutritional status, and related drugs. Four different models were considered: the Andersen and Gill model (AG model), the Williams and Peterson counting process model (PWP-CP model), the Williams and Peterson gap time model (PWP-GT model), and the frailty model.

There is no substantial difference in the risk of ASM-ADR recurrence between boys and girls, although the risk for girls is consistently lower than that for boys. Age plays a statistically significant role, with the risk of ASM-ADR recurrence decreasing as children grow older. According to the model used, the risk decreases by 5% to 76% for each additional year of life, with similar estimates of approximately 40% in the AG and PWP-CP models.

When seizure classification is considered, focal seizures are associated with a greater risk of ADR recurrence than are generalized seizures. However, this difference is statistically significant only in the AG, PWP-CP, and frailty models, with increased risks of 55%, 49% and 96%, respectively, although the confidence intervals suggest no clear distinction. Nutritional status at baseline is also a significant factor; specifically, severe malnutrition appears to have the lowest risk of ASM-ADR recurrence compared with the reference category of normal weight, with a risk reduction of approximately 50%. Additionally, according to the AG and PWP-CP models, mild malnutrition is associated with a lower risk than normal weight is, and according to the frailty model, obesity is also associated with a lower risk than normal weight is. Across all the models, mild and severe malnutrition exhibit similar risk values.

In terms of the drugs associated with ADRs, LEV was significantly different from the reference drug category VPA, with a greater risk of nonsevere ADR recurrence. The increased risk ranges from 145% to 263%, depending on the model used. Conversely, the frailty model indicates a lower risk of ADR recurrence under PHT, with a risk reduction of 48%.

Table 4 shows the risk factors associated with ASM-ADR recurrence events in patients with severe seriousness according to sex, age, seizure type, nutritional status, and related drugs. Four different models were considered: the AG model, the PWP-CP model, the PWP-GT model, and the frailty model.

In cases of severe ASM-ADRs, the patterns regarding the sex and age of the children are similar to those observed in nonsevere ADRs. There was no significant difference between boys and girls, and as age increased, the risk of ADR recurrence decreased. This decrease ranges from 2% to 68%, depending on the model used.

When considering seizure classification, the PWP-CP and PWP-GT models indicate that focal seizures have an approximately 30% lower risk of ADR recurrence than generalized seizures do. Moreover, the frailty model revealed that patients with focal seizures are at greater risk than those with generalized seizures. Importantly, this finding differs from the odds ratio (OR) in the frailty model for focal seizures.

Baseline nutritional status also plays a significant role in the risk of ASM-ADR recurrence. Specifically, according to the AG and PWP-GT models, obesity is associated with the highest risk of ADR recurrence compared with the reference category of normal weight, with an increase in risk exceeding 200%. In addition, according to the AG model, severe malnutrition is associated with a 32% lower risk of ADR recurrence than a normal weight.

With respect to the drugs associated with severe ADRs, LEV significantly reduced the risk, ranging from 39% to 62%, compared with the reference drug category, VPA. On the other hand, PHT presents a higher risk, with an increase ranging from 166% to 505%, depending on the model. Additionally, according to the AG model, OXC is associated with a lower risk of severe ADR recurrence, with a reduction of 55%.

For all the models, we conducted residual analyses to assess whether the underlying assumptions were satisfied. Overall, the analyses indicated a good model fit across all the cases. However, in the models focused on nonsevere recurrent events, few observations presented large residuals, suggesting potential outliers or deviations that may warrant further investigation.

## 3. Discussion

Patients with chronic medical conditions such as epilepsy receive long-term treatments that expose them to one or more ASMs for a long period of time. This ASM can cause recurrent ADRs, which cause increased morbidity and mortality, high medical costs and disruptions to patients’ lives. In many studies, researchers only include the initial event after starting treatment or after a change in dose or drug. However, it is possible to collect considerable information about patients while the treatment lasts. Unfortunately, large amounts of data concerning the temporal evolution of the disease or the effects of treatment are lacking. In our case, we assessed hospitalized pediatric epilepsy patients and followed them for 122 months. During the initial analysis of Hernandez García, 2023, a high percentage of children experienced recurrent adverse events [9]. Therefore, to account for temporal information about the recurrent presentation of ADRs in these children, we employed statistical models to identify risk factor profiles for ASM-ADR recurrence in pediatric patients with epilepsy. The several models used allowed us, on the one hand, to compare different categorical variables and observe their independent influence on the occurrence of ADRs (Kaplan–Meier method) and, on the other hand, to quantify the influence of several explanatory variables to model their risk on the recurrence of ADRs (AG model, PWP–CP model, PWP–GT model and frailty model).

Among the 313 children, 211 experienced some ASM-ADRs during the study, and 102 patients had no recurrent ASM-ADRs; notably, some patients had up to 9 ADRs. Building on our prior research [9], who identified both nonsevere (e.g., drowsiness, irritability, alopecia, erythema and constipation) and severe (e.g., thrombocytopenia, metabolic acidosis, hyperammonemia, liver damage and uncontrolled seizure) ASM-ADRs in hospitalized pediatric patients, and acknowledging that severe ADRs pose life-threatening risks, we conducted a separate analysis of ADR seriousness. This approach aimed to identify risk factors specific to severe ASM-ADRs, as preventing their recurrence could save lives, and these risk factors may differ from those associated with nonsevere events.

Interestingly, we observed that the majority of ASM-ADRs (70.5%) of both nonsevere and severe cases occurred during the first year of treatment. The remaining nonsevere ASM-ADRs occurred during the first 6 years, whereas severe ASM-ADRs can occur up to the ninth year of treatment. This could be explained by the fact that ASM-ADRs can present with different timings according to the type of reaction: (i) acute early idiosyncratic drug reactions associated with allergic mechanisms, (ii) dose-dependent action, and (iii) chronic phase adverse effects observed after long-term use [16]. Most ASM-ADRs due to idiosyncratic reactions occur between 1 and 2 weeks and between 2 and 3 months after the start of treatment. On the other hand, dose-related reactions usually occur with the first dose, after a subsequent dose increase, or after the addition of another drug [17]. Chronic ADRs can appear a few months to several years later due to prolonged exposure to drugs or unknown biochemical mechanisms [18]. However, it cannot be ruled out that some of the severe ADRs observed after several years of treatment may be due to dose changes, the addition of another drug or idiosyncratic reactions.

To provide a nonparametric estimation of the probability for the initial event to ASM-ADR, we used the Kaplan–Meier estimator [19], which independently showed that the variables with significant differences for ASM-ADR were age and nutritional status at the start of treatment and drug-related to the ADR. Compared with other age groups, infants under 1 year of age were more likely to present both nonsevere and severe ADRs. Nutritional status differentially affects the probability of presenting with ASM-ADR; children with mild malnutrition and obesity have a lower probability of presenting with nonsevere ADRs, whereas those with severe and mild malnutrition have a lower probability of presenting with severe ADRs. Furthermore, the type of medication prescribed for each patient had a significant effect on the probability of presenting ADRs. VPA, LEV, and PHT have a greater risk of presenting nonsevere ADRs, with similar probabilities; however, PHT has the highest risk of presenting severe ADRs, followed by VPA, with the second highest risk, and LEV, with the third highest risk of presenting severe ADRs.

The Kaplan–Meier estimator allows us to study only the effect of one factor at a time [19]; however, children with epilepsy are a mixture of characteristics that occur simultaneously. To provide a multivariate model of the risk factors that generate recurrent ASM-ADRs in these children, we used four survival models for recurrent events. We identified two profiles of variables that increase the risk of developing nonsevere and severe recurrent ASM-ADRs (in each case, the reference categories for the variables were seizure type generalized, nutritional status normal weight, and drug-related VPA): (1) Children at higher risk of developing nonsevere recurrent ADRs were those infants under 1 year of age, diagnosed with focal seizures, and treated with LEV. Surprisingly, in this group, children treated with VPA or PHT had similar risk factors for nonsevere ADRs, and severe malnutrition was a protective factor. (2) Children at greater risk of developing severe recurrent ADRs were those who were younger than 1 year of age, were obese and were treated with PHT. In this group of children, treatment with LEV may be a protective factor.

To understand the combination of risk factors, we must consider that the selection of ASM is closely related to the type of seizure diagnosed, especially when initiating treatment for patients with epilepsy [20]. The ILAE has classified seizure types based on the patient’s onset symptoms: focal-onset seizures, generalized-onset seizures, and unknown-onset seizures [5]. Based on this classification, physicians should select the appropriate ASM or combination of drugs that best control seizures and have the fewest adverse effects [21]. In 2023, the World Health Organization issued updated recommendations for the treatment of generalized-onset seizures (monotherapy with lamotrigine, LEV, or VPA, and PHT or phenobarbital as a second option) and focal-onset seizures (LEV or lamotrigine, carbamazepine or lacosamide) [22]. VPA is the recommended first-line drug for treating unknown epilepsies [23]. Following these recommendations, the drugs most commonly prescribed in Mexico to treat pediatric patients diagnosed with any of the three types of seizures are VPA, LEV, PHT, and OXC [9,24].

Each ASM has its own pharmacokinetic profile, and factors such as age or nutritional status can modify it and induce the appearance of ADRs. After an ASM is administered, its serum concentration is determined by one of four basic processes: absorption, distribution, metabolism, and excretion. Absorption involves the initial amount of drug in the body and influences its pharmacodynamics and adverse effects. On the other hand, distribution in the body is characterized by the volume of distribution (Vd), which is the theoretical volume occupied by the drug in the body. The third step, drug metabolism, includes all the chemical biotransformation reactions that lead to active, inactive, or toxic metabolites. While the last step is the excretion of the drug from the body, this step is frequently urinary or fecal [25,26].

When the pharmacokinetic profiles of VPA, LEV, and PHT were compared to understand why LEV is associated with non-severe ADRs and PHT with severe ADRs, significant differences were evident. VPA and PHT strongly bind proteins, and clinically significant changes in drug effects result from changes in the free fraction of the drug. On the other hand, LEV has little interaction with proteins and other drugs, resulting in more stable plasma concentrations [20]. With respect to its metabolism, LEV is metabolized primarily by enzymatic hydrolysis, and its metabolites have no pharmacological activity. On the other hand, VPA and PHT are metabolized in the liver. PHT is metabolized by cytochrome P450, generating inactive metabolites. Therefore, drugs that alter the function of this enzyme can induce or inhibit PHT. VPA, for example, inhibits cytochrome P450 and increases plasma concentrations of PHT, leading to overdose. The main route of VPA metabolism is microsomal glucuronide conjugation, which produces metabolites with anticonvulsant activity, whereas others exhibit toxicity. The half-life also differs: LEV has a half-life of 7 h, VPA has a half-life of 9–18 h, and PHT can last up to 40 h [20,27,28,29,30]. Considering the above findings, we can assume that LEV increases the risk of nonsevere ADRs compared with VPA because LEV has more stable plasma concentrations, its metabolites have no physiological activity, and its half-life is shorter. In contrast, PHT may cause more severe ADRs than VPA does because, owing to its kinetics, small changes in dose can cause disproportionate changes in its serum concentration, resulting in toxicity [31]. Furthermore, owing to its strong protein binding ability, reduced serum albumin for any reason (such as pregnancy, malnutrition, and liver disease) can increase the free fraction of PHT, which can remain in the body for a long time because of its long half-life, leading to an increased risk of adverse effects [27,32].

Interestingly, children’s age is a determining factor in the recurrence of both nonsevere and severe ASM-ADRs, as observed by the significance of the four hazard models used in this work. This may be because the pediatric population, especially in the neonatal and infant stages of development, presents dynamic changes in their physiology [25]. Ontogenetic morphological changes and changes in metabolism have profound effects on the pharmacokinetics and pharmacodynamics of antiepileptic drugs, especially during the first 18 months of life [25]. In infants and children, the gastric emptying time is shortened, and splanchnic blood flow is increased, leading to faster drug absorption and higher peak concentrations. When drugs are absorbed more rapidly, the variation between the maximum and minimum serum concentrations of the drug is greater, which can lead to more adverse effects [33]. Another change that occurs in pediatric pharmacokinetics during the early stages of development is their ability to metabolize drug molecules [34]. In pediatric patients, the production of metabolites in quantities sufficient to cause adverse effects has been observed because there is a more rapid transformation of the original drug to a metabolite, exceeding the capacity to eliminate it, resulting in its accumulation [33]. Some drug-metabolizing enzymes are not active until a certain age, resulting in drug accumulation. However, at other ages, there appears to be greater metabolic capacity, which may result in increased production of metabolites that are toxic to some drugs [35]. Examples of this include VPA-induced hepatotoxicity; hypersensitivity reactions to ASM, such as carbamazepine, PHT, phenobarbital and primidone; and lamotrigine-induced skin reactions [33]. Therefore, the intrinsic changes in children’s development and maturation make the first years of life a risk factor for a greater number of adverse effects, both nonsevere and severe, than in children whose maturation processes have reached limits such as those of adults. Our data support this theory, showing that the risk of developing recurrent ASM-ADR decreases in children each year of life, such that during the first year, the risk is more than 90%, and in adolescents, it is approximately 35%. Although this factor cannot be modified, treating physicians must be about its relevance when choosing antiepileptic treatment to avoid recurrent ASM-ADRs.

Nutritional status can affect the body’s response to drugs; malnutrition and obesity cause multiple physiological changes. The therapeutic response is more difficult to predict with respect to malnutrition status because patients with nutritional deficiency have more complicated problems, such as hypoalbuminemia and macronutrient and micronutrient deficiencies, which affect the levels of ASM [36]. Obesity can be characterized by an increase in blood flow and gastrointestinal transit, changes in body composition, hepatomegaly, and liver and kidney disorders. These pathophysiological differences lead to changes in drug pharmacokinetics that can significantly affect dosage, clinical tolerance, and efficacy [26]. In our case, we observed that severe malnutrition was associated with a risk reduction of approximately 50% for nonsevere ASM-ADR recurrence compared with the reference category of normal weight. According to only two models (AG and PWP-GT models), obesity is associated with a 200% greater risk of severe ASM-ADR than the reference category of normal weight. These effects are difficult to explain since previous studies have reported controversial results.

Hypoalbuminemia is a common characteristic of severe malnutrition and is characterized by a decrease in the level of albumin in plasma, which in turn increases the Vd of some drugs, such as VPA [37]. In malnourished pediatric patients, an increase in the free VPA concentration compared with that in healthy children was observed; however, no correlation was detected between elevated free serum VPA concentrations and ADRs [37]. Hypoalbuminemia also reduces the protein binding of PHT in plasma, and both the ratio of free/total PHT concentration and the total PHT concentration may be altered [38,39]. Ramasamy et al. (2010) reported that the concentration of free PHT in epileptic patients with malnutrition was 127% greater than that in normal nutrition patients [39]. In contrast, Bano et al. (1985) reported that the absorption kinetics, peak concentration and time to reach peak concentration in malnourished children treated with PHT did not significantly differ from those in the control group [40]. On the other hand, some works have focused on discovering the effects of obesity on the pharmacokinetics of ASM. In pediatric patients with obesity treated with fosphenytoin, no significant differences were found in the serum concentrations of PHT or its free fraction [41,42]. Similarly, Prusakov et al. (2018) reported that the total levels and Vd of PHT were similar between pediatric patients with and without obesity [43]. In terms of the adverse effects observed in obese patients treated with PHT, there are no significant differences in the type or frequency of adverse events between obese children and adults and normal-weight patients [42,44]. These reports suggest that while there are modifications in some pharmacokinetic parameters of ASM, this is not reflected in the presence of more ADRs in pediatric epileptic patients with malnutrition or obesity. This could obviate the need to consider nutritional status when prescribing ASM. However, as we mentioned before, a child with epilepsy is the sum of all their characteristics; with our models, we found that this characteristic is relevant for the development of recurrent ASM-ADRs.

Despite the strengths and merits of our study, there are several limitations that may impact the interpretation of ADR recurrence risk factors. First, intensive pharmacovigilance during the hospitalization period is restricted because ADRs that may occur after medical discharge are underreported, making outpatient follow-up necessary to record the full spectrum of events and determine the real frequency of ADR recurrence in long-term treatment. This could be achieved from logbooks completed by patients or their caregivers. Second, treatment modifications or ASM switching during the follow-up were not modeled as time-varying covariates, potentially confounding recurrence estimates owing to unaccounted changes in ASM type or dosage. Third, dynamic clinical variables such as dose changes, the appearance of comorbidities, concomitant medications, drug interactions, or changes in nutritional status during follow-up were not incorporated and may introduce residual confounding by influencing ADR risk in pediatric patients with complex medical profiles. In some cases, this was because such data were not recorded in the clinical files or because there was no actual monitoring of a variable. Furthermore, the study did not include direct pharmacokinetic measurements or biomarkers that would allow for pathophysiological validation of the findings related to the mechanisms of action or toxicity. Because the study was conducted in a tertiary care hospital, the generalizability of the results to other levels of care or settings with lower diagnostic capacity may be limited. Finally, the sample size of some specific subgroups, such as children under one year of age or patients with obesity, may affect the accuracy of risk estimates in these clinical profiles.

Finally, in our previous study [9], we conducted a descriptive analysis of ADRs, focusing on their characteristics. However, that study did not incorporate the temporal dimension or the recurrence of events. In contrast, the present work explicitly integrates time by modeling the repetition of ADRs over 122 months period via survival models for recurrent events. This temporal component represents a major contribution, as it allows us not only to identify which patients are at higher risk of ADRs but also to estimate when these events are most likely to recur. Thus, this study offers a more powerful clinical tool, enabling targeted monitoring and prevention strategies on the basis of the dynamic risk profile of each patient. Therefore, this second study complements and expands upon the original findings by providing a longitudinal and predictive perspective that enhances our understanding of ADR safety patterns in pediatric epilepsy treatment. 

## 4. Materials and Methods

### 4.1. Study Setting and Design

This was a cohort, prospective, observational, longitudinal, and descriptive study carried out in the neurology hospitalization area at the National Institute of Pediatrics (INP) from October 2012 to December 2022, Mexico city, Mexico.

### 4.2. Patients

Pediatric patients (from 0 to 18 years of age) with a diagnosis of epilepsy (given or confirmed by INP neurologists) and hospitalized at the INP were included. The patients had complete medical records, and their treatment, either as monotherapy or polytherapy, included one of the following ASM: LEV, VPA, PHT, OXC, TPM, clonazepam, carbamazepine, gabapentin, or clobazam. It should be noted that during the study, the physician made the adjustment of the doses and made the monitoring of the plasmatic levels of the VPA, PHT, and OXC until they obtained the therapeutic effects.

### 4.3. General Procedure and Recording of Adverse Drug Reactions

The study began with the identification of eligible patients who were invited to participate. If the parents or guardians and the patients agreed and met the inclusion criteria, they were included in the study.

First, patient data, such as age, sex, weight, height, seizure type, prescribed medications, and dose at the beginning and end of treatment, were collected. Subsequently, daily patient visits were made, and when a suspected ADR was detected, it was recorded in a specially designed format, and the treating physician was notified. The ADR was only recorded during the patients’ hospitalization. ADRs were recorded via the intensive pharmacovigilance method. The basis of this monitoring is a noninterventional observational cohort, which monitors only selected medications over a specific time period [45].

### 4.4. Instruments

The causal relationship between the ASM and the ADR was determined by applying the Naranjo algorithm [46,47]. To classify the seriousness of the ADR clinical manifestations (severe or nonsevere), the Mexican official standard NOM-220-SSA1-2016 (NOM-220-SSA1-2016) was used [15]. Severe ADRs were considered if they were medically important, endangered the patient’s life at the time of occurrence, required hospitalization or a prolonged hospital stay, or caused death. Nonsevere cases were those that did not meet the above criteria.

### 4.5. Ethical Considerations

The study was conducted in accordance with the Declaration of Helsinki. The protocol was approved on 26 September 2012 by the Ethics and Investigation Committees of the INP (register number 090/2012). All parents or guardians and patients were informed about the study, objectives, potential benefits, and potential discomforts. The protection of patients’ well-being, dignity, integrity, and privacy was guaranteed at all times. Informed consent forms were obtained and signed by all parents or guardians. Patients over 12 years of age signed informed consent forms. Patients (or their parents) were free to withdraw from this study at any time.

### 4.6. Definition of the Variables

To analyze the recurrence times of patients who presented with ADRs, survival models for recurrent events were used [48,49]. To do this, the target variables were first defined: recurrent event, recurrence time, and censoring. A recurrent event is defined as one in which “one or more ADRs occur in one day”, also called recurrences, taking into account both the classification of ADR seriousness (nonsevere, severe, or ADR absence) and the drug causing the ADR (VPA, LEV, PHT, OXC, TPM, or other). This means that if, in one day, the patient presents one or more ADRs, having the same type of ADR seriousness and having the same drug caused the ADR, then it is recorded that an event occurred on that day.

For each subject, the initial time (time = 0) is considered the date they begin the treatment. Therefore, the recurrence time is the time, measured on a scale of years, that elapses to the date on which the event occurs. Additionally, all the observed subjects who were included in this study and who did not present ADRs were considered censored. Therefore, for each subject, there are observed times and the status that identifies whether they are ADR nonsevere recurrence times (status = “ADR Nonsevere”), ADR severe recurrence times (status = “ADR Severe”), or censored times (status = “Absence”). Note that for censored data, the recurrence time, or observed time, is the time that elapses to the last date they were observed, hospitalized and included in the study. The explanatory variables were sex, age (at study entry), seizure type, and nutritional status.

### 4.7. Descriptive Analysis

A descriptive analysis of the observed times and number of recurrences of ADRs was performed separately for each of the explanatory variables. Specifically, tables and graphs showing the distributions of the number of events by sex, age (at study entry), seizure type, therapy, and nutritional status were generated.

### 4.8. Statistical Analysis of Recurrent Events

Kaplan–Meier survival analysis was used to estimate the time to the first event, providing a nonparametric description of survival probabilities over time. However, this method does not account for recurrent events or intra-individual correlations. Therefore, its use is limited to a descriptive purpose for the initial event and may lead to an underestimation of the total event burden over time [50].

For the statistical analysis of recurrent events, four different models were considered:

1. The AG model is an extension of the proportional hazard Cox regression model and uses the time interval of the counting process [51]. It assumes that recurrent events within subjects are independent and that they share a common reference risk.
2. The PWP-CP model is based on the counting process model [52]. It assumes that recurrent events within the subject are related and that the reference risk varies from one event to another. It evaluates the effect of a covariate for the kth event since the entry time in the study.
3. The PWP-GT model is based on the counting process model and is defined in terms of gap time [52]. It evaluates the effect of a covariate for the kth event since the time of the previous event.
4. The frailty model is another extension of the proportional hazard Cox regression model [53]. It assumes that the correlation between recurrent events is due to the tendency of some individuals to be more prone to developing recurrent events than others are due to unobserved or unknown factors, such as genetic factors.

To determine the risk factors associated with the target variables, the ORs, 95% confidence intervals, and associated *p* values were estimated.

For each model, the target variables are the observed times and the status of ADR recurrence by ADR severity. The linear predictor includes explanatory variables, i.e., sex (ref: boys vs. girls), age (at study entry, continuous scale), seizure type (ref: generalized vs. focal, unclassified), nutritional status (ref: normal weight vs. severe malnutrition, mild malnutrition, obesity), and drug-related factors related to ADRs (ref: VPA vs. LEV, PHT, OXC, TPM, other, or absence of ADR).

Statistical analysis was performed via R version 4.1.1. and RStudio version 1.4.1717 software, with the “stats”, “dplyr”, “ggplot2” [54], and “survival” packages [55,56]. A *p*-value < 0.05 was considered statistically significance.

## 5. Conclusions

In conclusion, in this work, we demonstrated that the application of survival models for recurrent events allows us to obtain risk factor profiles for the recurrence of ASM-ADRs in pediatric patients. Age, drug type, and nutritional status are the variables to consider when individualizing antiepileptic treatments to reduce the presence, severity, and recurrence of ADRs that limit the quality of life of these children.

## Figures and Tables

**Table 1:** Frequency of patients by number of ASM-ADR recurrence events for each explanatory variable.

Variable	Number of ASM-ADR Recurrences													
	0	1	2	3	4	5	6	7	8	9	10	11	12	Total
**Sex**														
Ref: Boys	59	47	31	15	6	5	4	3	2	0	1	1	1	**175**
Girls	43	44	24	14	6	2	3	0	2	0	0	0	0	**138**
**Age**														
Infant (1 m and <1 yr)	21	23	17	11	4	1	2	1	1	0	0	0	1	**82**
Older infant (1 yr and <2 yr)	15	12	11	2	1	0	1	0	0	0	1	0	0	**43**
Preschool (2–4 yr 11 m)	16	18	11	9	4	2	3	0	0	0	0	1	0	**64**
School-aged (5–9 yr 11 m)	17	22	7	4	2	2	0	1	2	0	0	0	0	**57**
Ref: Adolescent (10–18 yr)	33	16	9	3	1	2	1	1	1	0	0	0	0	**67**
**Seizure type**														
Ref: Generalized	48	46	31	15	4	2	3	0	3	0	0	0	1	**153**
Focal	32	37	21	12	8	4	3	2	1	0	1	1	0	**122**
Unclassified	22	8	3	2	0	1	1	1	0	0	0	0	0	**38**
**Therapy**														
Ref: Monotherapy	72	60	24	8	3	4	3	0	0	0	0	1	0	**175**
Polytherapy	30	31	31	21	9	3	4	3	4	0	1	0	1	**138**
**Nutritional status**														
Severe malnutrition	45	29	23	11	5	0	2	1	1	0	0	0	0	**117**
Mild malnutrition	16	23	9	3	4	0	0	0	1	0	0	0	0	**56**
Ref: Normal weight	31	33	22	14	2	7	5	1	2	0	1	1	0	**119**
Obesity	10	6	1	1	1	0	0	1	0	0	0	0	1	**21**
**Total**	**102**	**91**	**55**	**29**	**12**	**7**	**7**	**3**	**4**	**0**	**1**	**1**	**1**	**313**

ASM-ADRs: Anti-seizure medication-Adverse drug reactions; Ref: reference category; m: months, yr: years.

**Table 2:** Frequency of cases of ADR seriousness by recurrence time in years.

ASM-ADR	0–1 yr	1–2 yr	2–3 yr	3–4 yr	4–5 yr	5–6 yr	6–7 yr	7–8 yr	8–9 yr	9–10 yr	Total
Absence of ADR	77	13	3	4	2	1	1	0	1	0	**102**
ADR Nonsevere	186	43	10	9	3	1	1	0	0	1	**254**
ADR Severe	166	34	16	11	3	4	3	1	3	4	**245**
**Total**	**352**	**77**	**26**	**20**	**6**	**5**	**4**	**1**	**3**	**5**	**499**

ASM-ADRs: Anti-seizure medication-adverse drug reactions; yr: years.

**Table 3:** Analysis performed by the recurrent events models to analyze factors associated with ADR recurrence for nonsevere seriousness.

Variable	AG Model		PWP-CP Model		PWP-GT Model		Frailty Model	
n = 601, 102 censors	OR	(95% CI)	OR	(95% CI)	OR	(95% CI)	OR	(95% CI)
254 ADR Nonsevere								
**Sex**								
Ref: boys	1		1		1		1	
girls	0.848	(0.585, 1.229)	0.842	(0.582, 1.218)	0.911	(0.693, 1.198)	0.875	(0.617, 1.240)
**Age**								
Continue scale	0.560	(0.437, 0.718) *	0.603	(0.483, 0.753) *	0.951	(0.923, 0.981) *	0.244	(0.204, 0.292) *
**Seizure type**								
Ref: Generalized	1		1		1		1	
Focal	1.548	(1.113, 2.155) *	1.493	(1.045, 2.132) *	1.002	(0.782, 1.285)	1.961	(1.365, 2.817) *
Unclassified	1.916	(0.968, 3.791)	1.904	(0.906, 4.005)	1.051	(0.605, 1.824)	1.325	(0.652, 2.692)
**Nutritional status**								
Severe malnutrition	0.448	(0.301, 0.666) *	0.498	(0.332, 0.747) *	0.627	(0.456, 0.861) *	0.610	(0.413, 0.899) *
Mild malnutrition	0.469	(0.278, 0.790) *	0.573	(0.346, 0.948) *	0.704	(0.481, 1.030)	0.685	(0.420, 1.116)
Ref: Normal weight	1		1		1		1	
Obesity	0.915	(0.603, 1.389)	1.157	(0.630, 2.127)	1.341	(0.768, 2.342)	0.467	(0.219, 0.994) *
**Related drug**								
Ref: VPA	1		1		1		1	
LEV	1.865	(1.243, 2.800) *	2.630	(1.713, 4.040) *	2.085	(1.550, 2.803) *	1.450	(1.024, 2.052) *
PHT	1.185	(0.585, 2.401)	1.307	(0.643, 2.657)	1.079	(0.657, 1.771)	0.517	(0.309, 0.865) *
OXC	0.542	(0.241, 1.219)	0.454	(0.204, 1.010)	0.699	(0.280, 1.743)	0.703	(0.346, 1.425)
TPM	0.511	(0.211, 1.236)	0.589	(0.284, 1.222)	0.862	(0.481, 1.542)	0.637	(0.270, 1.506)
Other	1.386	(0.725, 2.651)	1.347	(0.706, 2.570)	1.427	(0.909, 2.238)	1.216	(0.621, 2.382)
Frailty							76.07	*p* value < 0.001

ADRs: adverse drug reactions; OR: odds ratio; CI: confidence interval; Ref: reference category; * *p* value < 0.05; VPA: valproic acid; LEV: levetiracetam; PHT: phenytoin; OXC: oxcarbazepine; TPM: topiramate.

**Table 4:** Analysis performed by the recurrent events models to analyze factors associated with ADR recurrence for serious severe cases.

Variable	AG Model		PWP-CP Model		PWP-GT Model		Frailty Model	
n = 601, 102 censors	OR	(95% CI)	OR	(95% CI)	OR	(95% CI)	OR	(95% CI)
245 ADR Severe								
**Sex**								
Ref: boys	1		1		1		1	
girls	0.880	(0.599, 1.292)	0.919	(0.618, 1.367)	0.976	(0.760, 1.254)	0.910	(0.658, 1.259)
**Age**								
Continue scale	0.661	(0.538, 0.813) *	0.691	(0.555, 0.860) *	0.977	(0.950, 1.005)	0.320	(0.274, 0.375) *
**Seizure type**								
Ref: Generalized	1		1		1		1	
Focal	0.950	(0.651, 1.387)	0.688	(0.459, 1.032)	0.763	(0.584, 0.997) *	1.515	(1.081, 2.123) *
Unclassified	1.224	(0.558, 2.683)	0.792	(0.388, 1.618)	1.008	(0.573, 1.773)	1.231	(0.671, 2.256)
**Nutritional status**								
Severe malnutrition	0.682	(0.468, 0.993) *	0.796	(0.524, 1.209)	1.030	(0.783, 1.356)	1.181	(0.828, 1.683)
Mild malnutrition	0.560	(0.287, 1.089)	0.765	(0.406, 1.440)	0.987	(0.664, 1.468)	1.227	(0.782, 1.926)
Ref: Normal weight	1		1		1		1	
Obesity	1.726	(1.005, 2.964) *	1.278	(0.530, 3.081)	2.204	(1.250, 3.887) *	0.843	(0.426, 1.669)
**Related drug**								
Ref: VPA	1		1		1		1	
LEV	0.496	(0.282, 0.872) *	0.512	(0.278, 0.945) *	0.607	(0.385, 0.956) *	0.380	(0.243, 0.595) *
PHT	4.899	(2.680, 8.955) *	5.048	(2.740, 9.301) *	2.905	(2.110, 4.001) *	1.659	(1.173, 2.345) *
OXC	0.456	(0.242, 0.857) *	0.591	(0.298, 1.172)	0.721	(0.398, 1.307)	0.718	(0.356, 1.448)
TPM	0.463	(0.211, 1.012)	0.515	(0.245, 1.082)	0.869	(0.449, 1.682)	0.567	(0.264, 1.222)
Other	0.490	(0.199, 1.206)	0.240	(0.064, 0.905) *	0.553	(0.251, 1.219)	0.496	(0.205, 1.199)
Frailty							38.83	*p*-value = 0.062

ADRs: adverse drug reactions; OR: odds ratio; CI: confidence interval; Ref: reference category; * *p* value < 0.05; VPA: valproic acid; LEV: levetiracetam; PHT: phenytoin; OXC: oxcarbazepine; TPM: topiramate.

## Data Availability

The data presented in this study are available on request from the corresponding author. The data are not publicly available due to privacy or ethical restrictions.

## Abbreviations

The following abbreviations are used in this manuscript:

ADR(s)	Adverse drug reaction(s)
AG model	Andersen and Gill model
ASM	Anti-seizure medications
CI	Confidence intervals
ILAE	International League Against Epilepsy
INP	National Institute of Pediatrics
LEV	Levetiracetam
OR	Odds Ratio
OXC	Oxcarbazepine
PHT	Phenytoin
*p* value	Probability value
PWP-CP model	The Williams and Peterson counting process model
PWP-GT model	The Williams and Peterson gab time model
Ref	Reference category
TPM	Topiramate
Vd	Volume of distribution
VPA	Valproic acid
